# CeutaOPEN, individual-based field observations of breeding snowy plovers *Charadrius nivosus*

**DOI:** 10.1038/s41597-020-0490-y

**Published:** 2020-05-20

**Authors:** Luke J. Eberhart-Phillips, Medardo Cruz-López, Lydia Lozano-Angulo, Salvador Gómez del Ángel, Wendoly Rojas-Abreu, Marcos Bucio-Pacheco, Clemens Küpper

**Affiliations:** 1 Research Group Behavioural Genetics and Evolutionary Ecology, Max Planck Institute for Ornithology, Eberhard-Gwinner-Str. 5, 82319 Seewiesen, Germany; 2 Posgrado en Ciencias del Mar y Limnología, Universidad Nacional Autónoma de Mexico, Ciudad Universitaria, 04510 Ciudad de Mexico, Mexico; 3 Naturaleza y Cultura Internacional, General Topete S/N, Col. Los Guayparines, 85760 Álamos, Mexico; 4 Laboratorio de Biología Evolutiva, Centro Tlaxcala de Biología de la Conducta, Universidad Autónoma de Tlaxcala, Carretera Tlaxcala-Puebla Km. 1.5, 90070 Tlaxcala, Mexico; 5 Departamento de Información y Bibliografia Especializada, Facultad de Biología, Escuela de Biologia, Universidad Autonoma de Sinaloa, Culiacan, 80013 Sinaloa Mexico

**Keywords:** Sexual selection, Behavioural ecology, Conservation biology, Population dynamics, Evolutionary ecology

## Abstract

Shorebirds (part of the order Charadriiformes) have a global distribution and exhibit remarkable variation in ecological and behavioural traits that are pertinent to many core questions in the fields of evolutionary ecology and conservation biology. Shorebirds are also relatively convenient to study in the wild as they are ground nesting and often occupy open habitats that are tractable to monitor. Here we present a database documenting the reproductive ecology of 1,647 individually marked snowy plovers (*Charadrius nivosus*) monitored between 2006 and 2016 at Bahía de Ceuta (23°54N, 106°57W) – an important breeding site in north-western Mexico. The database encompasses various morphological, behavioural, and fitness-related traits of males and females along with spatial and temporal population dynamics. This open resource will serve as an important data repository for addressing overarching questions in avian ecology and wetland conservation during an era of big data and global collaborative science.

## Abstract (summary)

**Table:** 

Measurement(s)	morphometric parameter • movement behavioral quality • offspring survival measurement • parental behavior • sexual interaction trait • social behavior
Technology Type(s)	fieldwork
Factor Type(s)	year of data collection • date of data collection • time of data collection • location of data collection • individual identity • sex of individual • family identity • nest initiation date • nest hatch date
Sample Characteristic - Organism	Charadrius nivosus
Sample Characteristic - Environment	saline pan • salt contaminated soil • intermittent saline evaporation pond • saline evaporation pond • saline marsh
Sample Characteristic - Location	Bahía de Ceuta, Sinaloa, Mexico

Machine-accessible metadata file describing the reported data: 10.6084/m9.figshare.12173511

## Background & Summary

Longitudinal data on individuals living in the wild represent the gold standard for research in organismal ecology, as subjects are sampled repeatedly over multiple stages of their life-history while being exposed to the natural evolutionary pressures of their native environments^1^. These types of data have offered evolutionary ecologists valuable insights into the selective processes that affect species over multiple generations such as, for example, the role of stochastic climate events shaping the beak morphologies of Darwin’s Finches^2^, the predator-prey cycles of mammal communities on the Serengeti^3^, or the demographic dynamics of alpine plants^4^ and animals^5^ in response to climate change. However, collecting field data over many consecutive years while following standardized methods requires substantial labour and consistent funding. Due to these challenges, raw longitudinal field data from wild populations are rarely made open to the public^6^ – thus limiting the transparency and reproducibility of published research methods and results in evolutionary ecology. Furthermore, releasing raw data has the potential benefit of stimulating more substantive discussion and criticism within the scientific community, which can advance research topics and forge productive collaborations. Here, we offer an open access database of our raw field observations over an 11-year period of 1,647 uniquely marked individuals from an important breeding population of snowy plovers (*Charadrius nivosus*) in Mexico.

*Charadrius* plovers are small ground-nesting shorebirds that occur worldwide. As a group, plovers present a model system for investigating fundamental and applied topics in organismal biology as they occupy open habitats that are easy to monitor and experimentally manipulate, and they exhibit intra- and interspecific variation in several behavioural, ecological, and demographic traits. For example, plovers display remarkable diversity and plasticity in breeding tactics with sex roles during courtship, mating, and parental care varying appreciably among populations both between and within species^7^. The snowy plover is native to North America^8^ and is one of the least abundant shorebirds on the continent (estimated population size: 25,869) with many populations in decline and requiring intensive management^9^. Apart from being a public icon of avian conservation, snowy plovers have also increasingly captured the spotlight for their intriguing ecology and life-history. Their unusual biology features a rare breeding behaviour characterized by highly dispersive polyandry and male-biased uniparental care^10,11^.

**Fig. 1:**
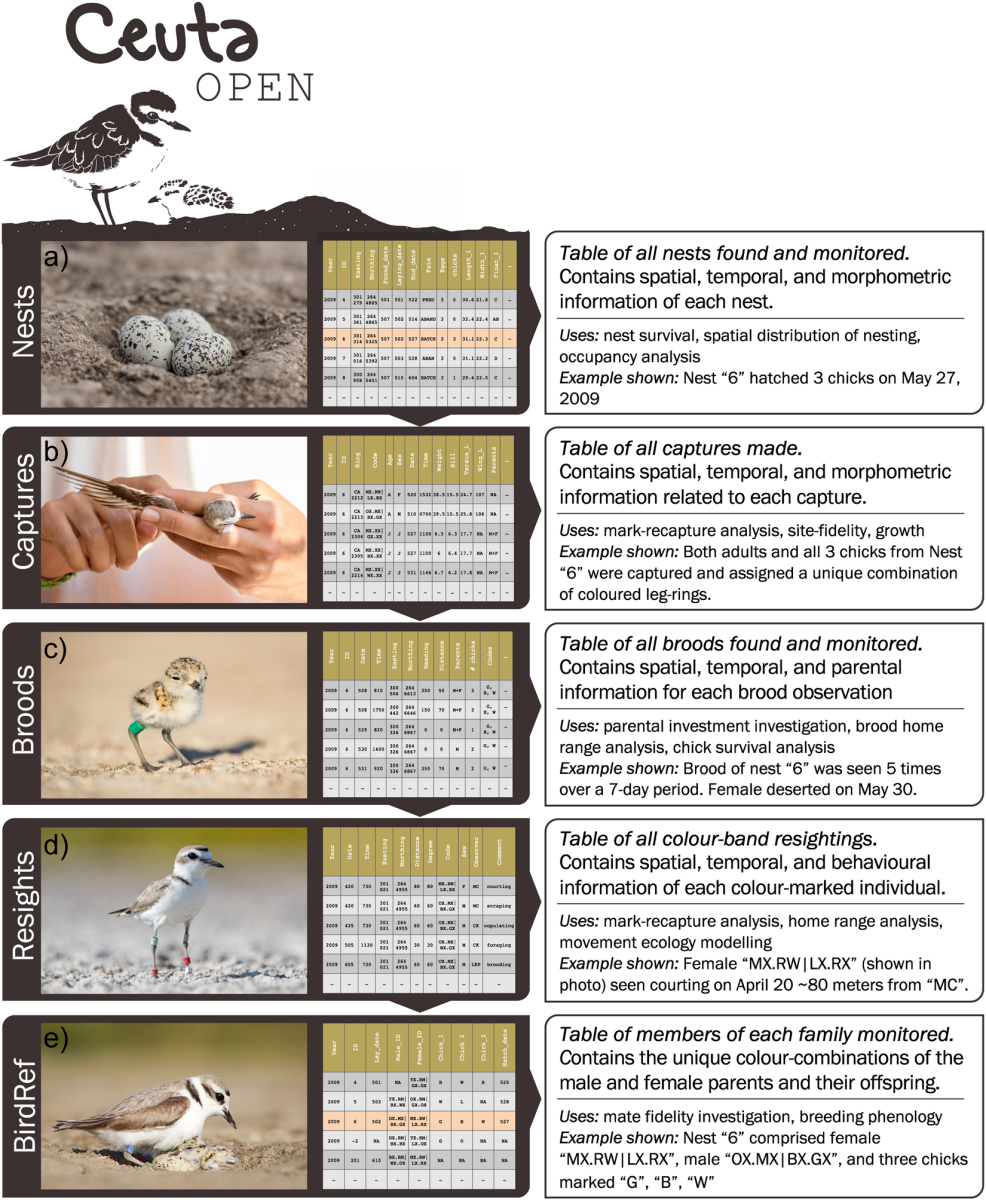
Schematic of the CeutaOPEN database. During fieldwork, data collection was divided across five main tasks: (**a**) nest monitoring, (**b**) captures of adults and chicks, (**c**) brood monitoring, (**d**) resightings of individually colour ringed adults, and (**e**) determining the identity of all breeding pairs and their offspring. The data obtained during each of these activities are structured in our database as the five tables shown here that contain a common variable such as a nest “ID” or a bird “code”, that can be utilized by the user for relational queries.

In this data descriptor we present CeutaOPEN – an open-access database containing the raw data from our fieldwork between 2006 and 2016 monitoring a breeding population of snowy plovers at Bahía de Ceuta, a subtropical lagoon on the coastal plain of north-western Mexico (23°54′N, 106°57′W). The database includes individual-based observations of reproductive effort, movements, morphometrics, and social behaviour (Fig. 1). Previously, we have used subsets of these data to report on a wide variety of topics in organismal biology, including sex ratio variation^12^, population viability^13^, courtship behaviour^14^, incubation behaviour^15^, parental care^16^, ontogeny^17^, chronobiology^18^, camouflage mechanisms^19^, offspring desertion^20^ and mating system dynamics^21^. The motivation for making our database open is to provide evolutionary ecologists with an accessible resource that will serve as an important repository for addressing overarching questions in organismal biology and conservation. Here we describe our field methods for collecting the observations presented in the database, we summarize the contents of the database, and we provide a code-based tutorial demonstrating how to import and query the database within the R environment and conduct, for example, a simple analytical workflow to investigate sex-specific ontogeny.

## Methods

### Study area

**Fig. 2:**
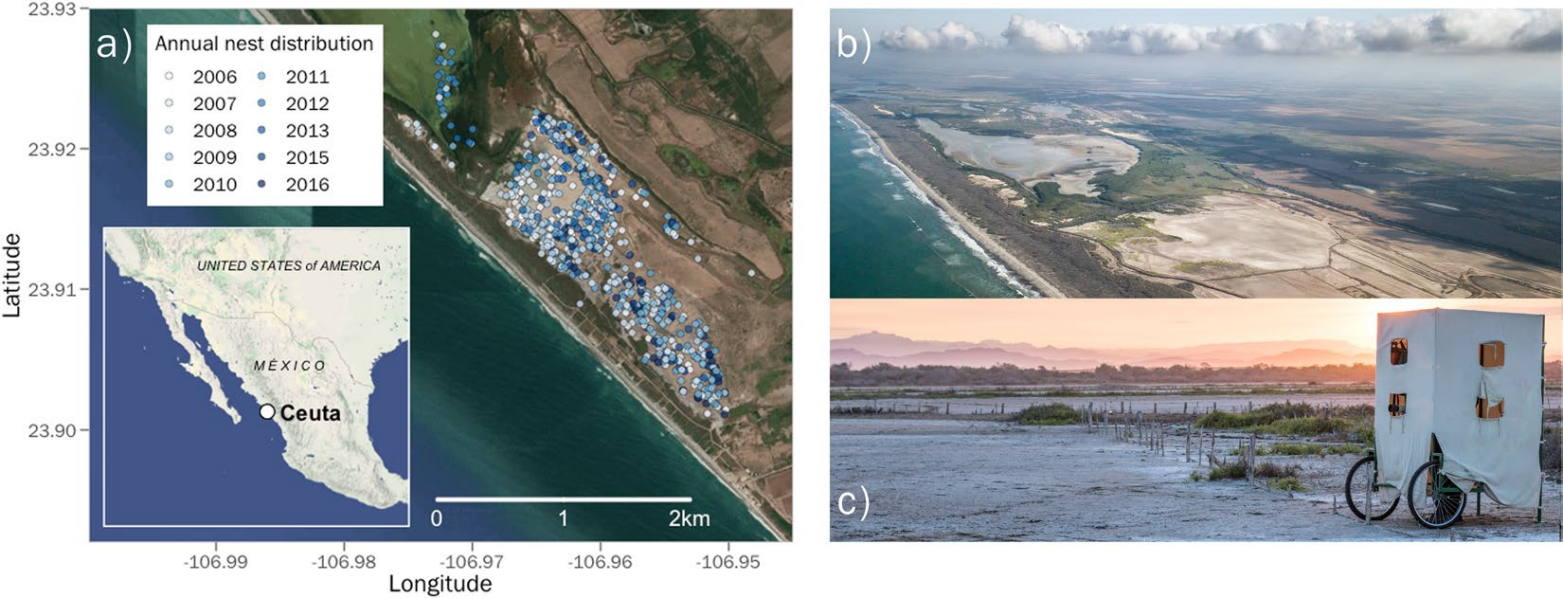
(**a**) Map of the Bahía de Ceuta study site and photos of the (**b**) salina breeding habitat and (**c**) a mobile hide in which observers conduct non-invasive field work.

Plovers breeding in Bahía de Ceuta mainly concentrate their activities on 200 ha of salt flats that contain several abandoned evaporation ponds. This habitat (hereafter “salina”) is surrounded by red mangrove (*Rhizophora mangle*) and characterized by sparse vegetation and open substrates. Nesting typically commences in late March or early April when flooding from spring tides and high precipitation recedes. By mid-July the breeding season concludes when rains and spring tides resubmerge the salina. Throughout the remainder of the year, the flooded salina and surrounding lagoons are used as important wintering habitats for plovers and other migratory shorebirds, with the region being protected by the Ramsar convention^22^. Our monitoring effort throughout the 11-year study period was focused on the largest contiguous section of salt flats in the study area where the vast majority of known breeding activity occured (Fig. 2a,b). However, in drought years or at the peak of the breeding season when tidewaters had maximally retreated, we made observations of plovers nesting and tending broods in several small pockets of salina adjacent to the main study site (Fig. 2a).

### Data collection

Over the 11-year study period, we monitored the population daily between April and July, and once every month or two during the remainder of the year. We used a car and mobile hides^23^ (Fig. 2c) to search for nests, broods, and determine the identity of breeding plovers with binoculars and scopes. During fieldwork, our data collection was divided across four main field tasks: (1) nest monitoring, (2) captures of adults and chicks, (3) brood monitoring, and (4) resights of individually colour ringed adults. The data obtained during each of these activities are structured in our database as tables (Fig. 1) containing a common variable such as a nest “ID” or a bird “code”, that can be utilized by the user for relational queries. The basic format of these tables was taken from ref. ^24^. Fieldwork permits to collect the data presented in CeutaOPEN were granted by the Secretaría de Medio Ambiente y Recursos Naturales (SEMARNAT). All of our field activities were performed in accordance with the approved ethical guidelines outlined by SEMARNAT. Here we explain the details of our data collection pertinent to the database.

#### Nest data

We regularly searched for nests (Fig. 1a) and incubating plovers by traversing the salina on foot, by car or in a mobile hide^19^ (Fig. 2c). Upon discovery, we recorded the nest’s geographic location, the found date and time, and measured the width and length of each egg in the clutch with calipers. To estimate the initiation-date of the clutch (i.e., date when the first egg was laid), we floated each egg in a jar of water and scored the embryonic stage of development according to a calibrated table^25^. For hatched clutches that were initially discovered more than 10 days after laying, we estimated initiation-date by subtracting 25 days (i.e., the mean incubation time in our population^18^) from the hatching date and subtracting an additional 5 days to account for a 2-day egg laying interval^11^. We checked nests every 2–7 days to assess survival and identify tending parents.

#### Capture data

**Fig. 3:**
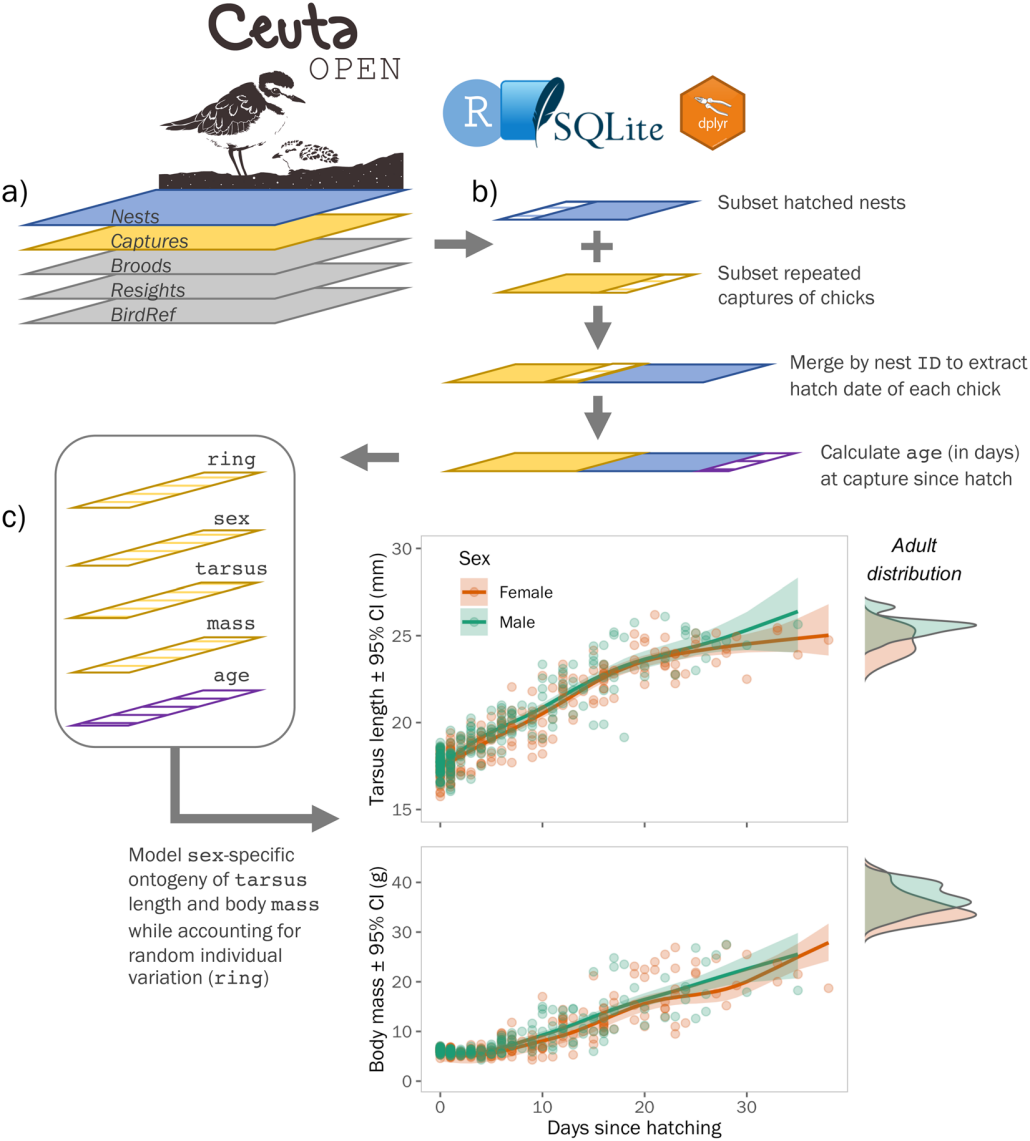
Schematic of an example analytical workflow using CeutaOPEN within the R environment to investigate how chick morphometric data may be used to study growth and ontogeny. (**a**) Import the database into R using the RSQLite^31^ package. (**b**) Use the dplyr^32^ package to join the ‘*Captures’* table with the ‘*Nests’* table by nest “ID” to determine the “hatch_date” of each captured individual. Subset the result to individuals that have repeated captures and calculate the “age” at capture by subtracting the “hatch_date” by the capture “date”. (**c**) Use the bamlss^33^ package (i.e., “Bayesian Additive Models for Location, Scale, and Shape”) to determine the sex-specific growth trends while controlling for repeated measures within individuals and random annual variation. Plot the fitted values to visualise the trends (see Supplementary File 1 for more details).

We captured plover chicks by hand and adults using mist nets or funnel traps on broods or nests. To individually identify members of the population, we assigned adults a unique combination of three to four colour leg rings and an alpha-numeric metal ring (see photo in Fig. 1d). Likewise, we marked chicks less than 2 weeks old with a single colour ring and a metal ring (see photo in Fig. 1c). Given our intensive nest search and capture efforts, we are confident that we ringed the vast majority of chicks (>95%) and breeding adults (>85%) in the local breeding population every year. During captures, we sampled the metatarsal vein of chicks or the brachial vein of adults and drew ∼25–50 μL of blood for subsequent genetic analyses. Additionally, we measured body mass, bill length, tarsus length, and wing length for all captured individuals (Fig. 3). As snowy plovers exhibit only minor sexual dimorphisim in plumage and body size, we molecularly determined sex using the Z-002B marker^26^ and verification with the Calex-31 marker located on the W chromosome^27^ for all adults and chicks captured before 2014. For PCR conditions see ref. ^17^.

#### Brood data

Similar to our data collection of nests, we resighted broods (see photo in Fig. 1c) every 1–7 days to assess chick survival and determine sex-specific patterns of parental care and desertion. Each brood observation includes the time, distance and azimuth from the observer to the brood, geographic location of the observer, number of chicks seen, and the identity of them and their parents.

#### Resight data

We typically resighted colour ringed individuals (see photo in Fig. 1d) opportunistically while in the field. Since 2009 we surveyed the entire salina within a single day at least once during the breeding season to record all colour ringed individuals present. As with our brood data, each resight includes the distance and azimuth to the individual, the geographic location of the observer, and any noteworthy comments pertaining to the individual’s behaviour.

## Data Records

Our database and all other files described in this manuscript are stored in a publicly available OSF repository^28^. The file Ceuta_OPEN_vX-X.sqlite contains the SQL (Structured Query Language) database of four tables containing our raw observations collected during routine fieldwork (Nests, Captures, Broods, and Resights), and a fifth table (BirdRef) that uses relational information to summarize the identities of the parents and offspring belonging to each nest and subsequent brood. The structure of these tables is defined in Tables 1–5 below. This Data Descriptor is based on version 1.4 of the CeutaOPEN database.

**Table 1:** Nests data of snowy plovers breeding in Bahía de Ceuta, Mexico, between 2006 and 2016.

Column	Data name	Description of data
1.	species	species of plover (all snowy plover ‘SNPL’ in this case)
2.	population	population at which nest was monitored (all ‘Ceuta’ in this case)
3.	year	year during which nest was monitored
4.	site	site at which nest was monitored
5.	nest	unique identifier of nest (unique within year and within site)
6.	ID	a concatenation of year, site, and nest to make a unique nest identifier across sites and years
7.	easting	UTM easting of nest
8.	northing	UTM northing of nest
9.	utm	UTM zone of nest
10.	found_date	date nest was discovered (stored in the internal ‘Date’ format of R and represents the number of days since January 1, 1970, the ‘Unix epoch’. Converted easily in R using ‘as.Date(found_date, origin = “1970-01-01”)’)
11.	found_time	time nest was discovered (24 h format, e.g. ‘1633’)
12.	nest_initiation_date	estimated date when the first egg of the nest was laid (i.e., its ‘initiation’). The estimate is calculated by subtracting the age in days of the oldest egg (determined by the floatation scores ‘float1’, ‘float2’, and ‘float3’ defined below) and a 5-day laying period for three-egg clutches or a 3-day laying period for two-egg clutches or a 1-day laying period for one-egg clutches (egg-laying intervals are based on ref. ^34^. Determining initiation dates of clutches found at stage ‘F’ is imprecise, and thus we estimated the initiation date by subtracting 25 days from the hatch date (i.e., the average length of incubation in this population) and an additional 5, 3, or 1 days for the laying period depending on the clutch size. For nests found at stage ‘F’ that failed before hatching, the nest initiation date is ‘NA’. Stored in the internal ‘Date’ format of R and represents the number of days since January 1, 1970, the ‘Unix epoch’. Converted easily in R using ‘as.Date(nest_initiation_date, origin = “1970-01-01”)’)
13.	end_date	date nest ended (cause specified in ‘fate’ column; stored in the internal ‘Date’ format of R and represents the number of days since January 1, 1970, the ‘Unix epoch’. Converted easily in R using ‘as.Date(end_date, origin = “1970-01-01”)’)
14.	last_observation_alive	date nest was last observed active. Stored in the internal ‘Date’ format of R and represents the number of days since January 1, 1970, the ‘Unix epoch’. Converted easily in R using ‘as.Date(last_observation_alive, origin = “1970-01-01”)’)
15.	fate	fate of nest (e.g., ‘Hatched’, ‘Predated’, ‘Abandoned’, etc.)
16.	male	colour-ring combination of male confirmed tending nest. The scheme can be noted as XX.XX|XX.XX where X indicates a possible position for a color (or metal) ring, the full-stop marks the position of the knee-joint and the pipe divides the left and right leg. Thus the readout is “left above. left below | right above. right below”. See page 9 of ref. ^25^ for more details.
17.	female	colour-ring combination of female confirmed tending nest. The scheme can be noted as XX.XX|XX.XX where X indicates a possible position for a color (or metal) ring, the full-stop marks the position of the knee-joint and the pipe divides the left and right leg. Thus the readout is “left above. left below | right above. right below”. See page 9 of ref. ^25^ for more details.
18.	no_chicks	number of chicks hatched from nest
19.	clutch_size	number of eggs found in nest
20.	length1	length in millimeters of egg #1
21.	width1	width in millimeters of egg #1
22.	float1	float score of egg #1 as defined on page 5 of ref. ^25^
23.	length2	length in millimeters of egg #2
24.	width2	width in millimeters of egg #2
25.	float2	float score of egg #2 as defined on page 5 of ref. ^25^
26.	length3	length in millimeters of egg #3
27.	width3	width in millimeters of egg #3
28.	float3	float score of egg #3 as defined on page 5 of ref. ^25^
29.	photo	indication if a photo of nest was taken (1) or not (0)
30.	observer	initials of observer who found nest
31.	comments	miscellaneous comments pertinent to nest’s observation

This dataset contains information on egg dimensions, laying phenology, nest fate, geographic location, and the identity of incubating parents. These data can be used to assess individual reproductive effort and success, mate and site fidelity, and senescence, for example.

**Table 2:** Captures data of snowy plovers breeding in Bahía de Ceuta, Mexico, between 2006 and 2016.

Column	Data name	Description of data
1.	species	species of plover (all snowy plover ‘SNPL’ in this case)
2.	population	population at which capture was made (all ‘Ceuta’ in this case)
3.	year	year during which capture was made
4.	site	site at which capture was made
5.	nest	unique identifier of nest at which capture was made (unique within year and within site). If capture was made at a brood originating from an unknown nest, the identifier is negative (e.g., ‘−2’).
6.	ID	a concatenation of year, site, and nest to make a unique nest identifier across sites and years
7.	ring	alpha-numeric code of metal ring assigned to captured individual
8.	code	color-ring combination assigned to captured individual. The scheme can be noted as XX.XX|XX.XX where X indicates a possible position for a color (or metal) ring, the full-stop marks the position of the knee-joint and the pipe divides the left and right leg. Thus the readout is “left above. left below | right above. right below”. See page 9 of ref. ^25^ for more details.
9.	age	age of captured individual (‘J’ = juvenile (chicks and first-years), ‘A’ = adult (second-years and older))
10.	field_sex	sex of individual determined in the field based on ornamentation and other clues (e.g., time of capture, parental care, etc.), where ‘F’ = female, ‘M’ = males, and ‘J’ = unknown sexed juvenile
11.	mol_sex	sex of individual determined in the lab with the P2/P8 and Calex-31 markers (for our PCR conditions see ref. ^17^), where ‘F’ = female, ‘M’ = males, ‘U’ = insufficient molecular evidence (e.g., markers failed), and ‘NA’ = individual not molecularly sex-typed. Note: all birds initially captured in years after 2013 have not yet been molecularly sex-typed
12.	sex	sex of captured individual (‘F’ = female, ‘M’ = males, ‘J’ = juvenile of unknown sex)
13.	easting	UTM easting of capture
14.	northing	UTM northing of capture
15.	utm	UTM zone of capture
16.	date	date capture was made (stored in the internal ‘Date’ format of R and represents the number of days since January 1, 1970, the ‘Unix epoch’. Converted easily in R using ‘as.Date(date, origin = “1970-01-01”)’)
17.	time	time capture was made (24 h format, e.g. ‘1633’)
18.	parents	parents attending captured individual (if age = ‘J’) at time of observation (‘0’ = no parent present; ‘1’ = one parent (not identified whether male or female); ‘2’ = female only (‘2+’ when female identified, whilst male’s identity was uncertain); ‘3’ = male only (‘3+’ when male identified, whilst female’s identity was uncertain, i.e., opposite of ‘2+’); ‘4’ = both present)
19.	weight	weight in grams of captured individual
20.	bill	length in millimeters of upper mandible of captured individual. Measured as the distance between the tip of the forehead feathering at the base of the upper bill, along the ridge of the culmen, and the tip of the bill (also known as the “exposed culmen” measurement; *sensu* page 8 of ref. ^35^
21.	left_tarsus	length in millimeters of left tarsus of captured individual. Measured as the distance between the notch at the end of the lateral condyle of the tibiotarsus on the backside of the leg, to the last tarsal scute on the front of the leg at the base of the foot (also known as the “outside tarsus” or “diagonal tarsus” measurement; *sensu* page 11 of ref. ^35^
22.	right_tarsus	same as ‘left_tarsus’ measurement above but for right leg of captured individual
23.	left_wing	length in millimeters of left wing of captured individual. Measured as the distance from the carpal joint (the bend of the wing) to the longest primary feather whilst flattening the wing and straightening the primaries (also known as the “maximum flat” or “flattened and straightened” measurement; sensu page 6 of ref. ^35^
24.	right_wing	same as ‘left_wing’ measurement above but for right wing of captured individual
25.	blood	indication if blood from captured individual was collected (‘1’) or not (‘0’)
26.	moult	primary molt score of captured individual. Scored as the stage of the moult and the number of feathers at that stage. See ref. ^36^ for more details.
27.	fat	fat score of captured individual, scored as the amount of visible fat in the furcular region or tracheal pit. See ref. ^36^ for more details.
28.	lice	indication if feather lice from captured individual were collected (‘1’) or not (‘0’)
29.	faecal	indication if faeces from captured individual was collected (‘1’) or not (‘0’)
30.	photo	indication if a photo of captured individual was taken (‘1’) or not (‘0’)
31.	observer	initials of observer making capture
32.	comments	miscellaneous comments pertinent to capture event

This dataset contains information on bird morphology, age, sex, capture time and location, and the identity of the individual. These data can be used to assess apparent survival with mark-recapture models, site fidelity, and growth rates of chicks, for example.

**Table 3:** Broods data of snowy plovers breeding in Bahía de Ceuta, Mexico, between 2006 and 2016.

Column	Data name	Description of data
1.	species	species of plover (all snowy plover ‘SNPL’ in this case)
2.	population	population at which brood was observed (all ‘Ceuta’ in this case)
3.	year	year during which brood was observed
4.	site	site at which brood was observed
5.	brood	unique identifier of brood (unique within year and within site). Broods originating from known nests retain the nest identifier found in the *Nests* table, whereas broods hatching from unknown nests have a negative identifier (e.g., ‘−2’)
6.	ID	a concatenation of year, site, and nest to make a unique brood identifier across sites and years
7.	easting	UTM easting of brood observation
8.	northing	UTM northing of brood observation
9.	utm	UTM zone of brood observation
10.	date	date brood observation was made (stored in the internal ‘Date’ format of R and represents the number of days since January 1, 1970, the ‘Unix epoch’. Converted easily in R using ‘as.Date(date, origin = “1970-01-01”)’)
11.	time	time brood observation was made (24 h format, e.g. ‘1633’)
12.	distance	estimated distance in meters between brood and observer
13.	degree	estimated bearing of brood relative to observer (i.e., the number of degrees in the angle measured in a clockwise direction from the north line to the line joining the observer to the brood)
14.	parents	parents attending brood at time of observation (‘0’ = no parent present; ‘1’ = one parent (not identified whether male or female); ‘2’ = female only (‘2+’ when female identified, whilst male’s identity was uncertain); ‘3’ = male only (‘3+’ when male identified, whilst female’s identity was uncertain, i.e., opposite of ‘2+’); ‘4’ = both present)
15.	male	color-ring combination of male observed tending brood. The scheme can be noted as XX.XX|XX.XX where X indicates a possible position for a color (or metal) ring, the full-stop marks the position of the knee-joint and the pipe divides the left and right leg. Thus the readout is “left above. left below | right above. right below”. See page 9 of ref. ^25^ for more details
16.	female	color-ring combination of female observed tending brood. The scheme can be noted as XX.XX|XX.XX where X indicates a possible position for a color (or metal) ring, the full-stop marks the position of the knee-joint and the pipe divides the left and right leg. Thus the readout is “left above. left below | right above. right below”. See page 9 of ref. ^25^ for more details
17.	chicks	number of chicks observed in brood. Because of temporary or permanent brood adoption, number of chicks can be larger than initial brood size at subsequent observations
18.	chick_codes	color ring combinations of all chicks observed (individuals seperated by a comma). The scheme can be noted as XX.XX|XX.XX where X indicates a possible position for a color (or metal) ring, the full stop marks the position of the knee-joint and the pipe divides the left and right leg. Thus the readout is “left above. left below | right above. right below”. See page 9 of ref. ^25^ for more details.
19.	brood_photo	indication if a photo of the brood was taken (‘1’) or not (‘0’)
20.	observer	initials of observer making brood observation
21.	comments	miscellaneous comments pertinent to brood’s observation

These data contains information on the time and location of a brood observation, the identity and number of chicks seen alive, and the identity of the parents tending chicks. These data can be used to assess parental investment, brood home range, and chick survival, for example.

**Table 4:** Resights data of snowy plovers breeding in Bahía de Ceuta, Mexico, between 2006 and 2016.

Column	Data name	Description of data
1.	species	species of plover (all snowy plover ‘SNPL’ in this case)
2.	population	population at which resighting was made (all ‘Ceuta’ in this case)
3.	year	year during which resighting was made
4.	site	site at which resighting was made
5.	easting	UTM easting of observer’s location while resighting
6.	northing	UTM northing of observer’s location while resighting
7.	utm	UTM zone of observer’s location while resighting
8.	date	date resighting was made (stored in the internal ‘Date’ format of R and represents the number of days since January 1, 1970, the ‘Unix epoch’. Converted easily in R using ‘as.Date(date, origin = “1970-01-01”)’)
9.	time	time resighting was made (24 h format, e.g. ‘1633’)
10.	distance	estimated distance in meters between resighted bird and observer
11.	degree	estimated bearing of resighted bird relative to the observer (i.e., the number of degrees in the angle measured in a clockwise direction from the north line to the line joining the observer to the brood)
12.	code	color-ring combination of the resighted individual. The scheme can be noted as XX.XX|XX.XX where X indicates a possible position for a color (or metal) ring, the full stop marks the position of the knee-joint and the pipe divides the left and right leg. Thus the readout is “left above. left below | right above. right below”. See page 9 of ref. ^25^ for more details.
13.	sex	sex of individual determined in the field based on ornamentation and other clues (e.g., capture history, parental care, etc.), where ‘F’ = female, ‘M’ = males, and ‘J’ = unknown sexed juvenile
14.	census	indication if the resighting was conducted as part of a census count (‘1’) or not (‘0’)
15.	observer	initials of observer making resighting
16.	comments	miscellaneous comments pertinent to the resighting

This dataset contains information on the time and location of a colour-ringed adult, the identity of the individual, and behavioural information recorded during the observation. These data can be used to assess apparent survival with mark-recapture models or investigate space-use through home range analysis or movement ecology models.

**Table 5:** Bird Reference (“BirdRef”) data of snowy plovers breeding in Bahía de Ceuta, Mexico, between 2006 and 2016.

Column	Data name	Description of data
1.	species	species of plover (all snowy plover ‘SNPL’ in this case)
2.	population	population at which family was observed (all ‘Ceuta’ in this case)
3.	year	year during which family was observed
4.	site	site at which family was observed
5.	family	unique identifier of family (unique within year and within site). Families found as nests retain nest identifier found in *Nests* table, whereas families found as broods hatching from unknown nests have a negative brood identifier (e.g., ‘−2’) found in *Broods* table)
6.	ID	a concatenation of year, site, and nest to make a unique family identifier across all sites and years
7.	nest_initiation_date	estimated date when the first egg of the nest was laid (i.e., its ‘initiation’). The estimate is calculated by subtracting the age in days of the oldest egg (determined by the floatation scores ‘float1’, ‘float2’, and ‘float3’ defined in the *Nests* table) and a 5-day laying period for three-egg clutches or a 3-day laying period for two-egg clutches or a 1-day laying period for one-egg clutches (egg-laying intervals are based on ref. ^34^. Determining initiation dates of clutches found at stage ‘F’ is imprecise, and thus we estimated the initiation date by subtracting 25 days from the hatch date (i.e., the average length of incubation in this population) and an additional 5, 3, or 1 days for the laying period depending on the clutch size. For nests found at stage ‘F’ that failed before hatching, the nest initiation date is ‘NA’. Stored in the internal ‘Date’ format of R and represents the number of days since January 1, 1970, the ‘Unix epoch’. Converted easily in R using ‘as.Date(nest_initiation_date, origin = “1970-01-01”)’)
8.	hatching_date	date nest hatched (stored in the internal ‘Date’ format of R and represents the number of days since January 1, 1970, the ‘Unix epoch’. Converted easily in R using ‘as.Date(hatching_date, origin = “1970-01-01”)’; ‘NA’ if nest fate was other than ‘hatch’ in *Nests* table)
9.	male	metal ring alpha-numeric code of male parent observed with nest/brood
10.	female	metal ring alpha-numeric code of female parent observed with nest/brood
11.	chick1	metal ring alpha-numeric code of first chick assigned to brood
12.	chick2	metal ring alpha-numeric code of second chick assigned to brood
13.	chick3	metal ring alpha-numeric code of third chick assigned to brood
14.	exp	indication if family was part of an experiment
15.	type	indication of type of experiment conducted
16.	manip	date of possible experimental manipulation (stored in the internal ‘Date’ format of R and represents the number of days since January 1, 1970, the ‘Unix epoch’. Converted easily in R using ‘as.Date(manip, origin = “1970-01-01”)’)

This dataset is a relational table of the *Nests*, *Captures*, and* Broods* tables (Tables 1, 2, and 3) summarizing the identity of all members in a family (i.e., metal ring alpha-numeric codes of both parents and all chicks, if applicable). These data can be used to quantify mating system and assess individual variation in breeding phenology, for example.

In summary, the CeutaOPEN database contains information on 794 surveyed nests, 2,824 captures of 1,647 marked individuals, 415 monitored broods, and 6,939 resightings of colour-marked individuals. Over the 11-year study period, we spent 927 days collecting these data in the field – amounting to over 20,000 hours of observational effort.

CeutaOPEN is one of only a few open-access databases to provide raw field observations of an individually-marked wild vertebrate species (for other examples, see refs. ^29,30^). We therefore believe our database will provide a valuable model for future field biologists to consult when structuring their data and deciding whether to provide public access.

## Technical Validation

During each field season of the snowy plover project at Bahía de Ceuta, observers receive comprehensive training on our sampling protocol^25^ and general avian field methodology. In all 11 years of data collection, at least one of us was present in the field to oversee fieldwork and assess the quality of observations. Moreover, field assistants usually aided us with fieldwork for academic purposes (e.g., as part of a bachelor, master, or doctoral project), which encouraged personal interest in maximizing the quality of their data collection. All breeding data from the 2014 breeding season was lost, which is why this year is missing the nest and brood data (Table 6). Likewise, 2015 does not include brood data because broods were not resighted in this year (Table 6).

**Table 6:** Annual summary in data of captures conducted, nests surveyed, broods monitored, and resights of colour-marked individuals.

Year	Captures	Nests	Broods	Resights
2006	456	158	69	19
2007	655	140	102	593
2008	229	76	30	577
2009	335	82	48	1257
2010	438	96	69	913
2011	225	70	35	806
2012	92	34	12	154
2013	87	31	16	491
2014	10	—	—	500
2015	148	50	—	812
2016	149	57	34	817

During the data processing and development of the final database, verification and validations were made at several stages: during fieldwork we would regularly check each other’s notes for unusual observations, during the digitization of field data in spreadsheets we would scrutinize outlier measurements, and throughout the assembly of the SQL database we conducted thorough data cleaning (e.g., removing white space from strings, enforcing consistent notation and symbology, etc.). These data quality checks were run annually before merging new observations with the master database.

## Usage Notes

The CeutaOPEN database is available under a Creative Commons Attribution 4.0 International Public License, whereby anyone may freely use and adapt our data, as long as the original source is credited, the original license is linked, and any changes to our data are indicated in subsequent use. The database has undergone multiple rounds of curation to purge inconsistencies and errors. Any further errors that are spotted by us or brought to our attention by users will be corrected and documented in future version releases of the database. When using any of the CeutaOPEN materials presented here, please cite this Data Descriptor in addition to the version of the database that was used. Furthermore, for all projects making considerable use of the CeutaOPEN database, we encourage users to reach out to us to offer the opportunity to comment prior to the publication of their work.

We recommend that users employ R to access and wrangle the CeutaOPEN database for their study. To help this process, please refer to the accompanying RMarkdown document (Supplementary File 1) to follow our suggested analytical workflow for utilizing CetuaOPEN with the RSQLite^31^ and dplyr^32^ packages in the R environment.

## Supplementary information

Supplementary File 1

## Data Availability

To assist users with accessing and querying our database, we have written an accompanying RMarkdown document (Supplementary File 1) that provides a commented workflow for utilizing CetuaOPEN with the RSQLite^31^ and dplyr^32^ packages in R.
